# Parental Reported Bullying among Saudi Schoolchildren: Its Forms, Effect on Academic Abilities, and Associated Sociodemographic, Physical, and Dentofacial Features

**DOI:** 10.1155/2020/8899320

**Published:** 2020-10-01

**Authors:** Raghad S. Alabdulrazaq, Sanaa N. Al-Haj Ali

**Affiliations:** ^1^College of Dentistry, Qassim University, Qassim, Saudi Arabia; ^2^Department of Orthodontics and Pediatric Dentistry, College of Dentistry, Qassim University, Qassim, Saudi Arabia

## Abstract

**Aim:**

To determine the prevalence of bullying, its forms, and its effect on academic abilities and school attendance, as well as associated sociodemographic, physical, and dentofacial features among Saudi schoolchildren.

**Methods:**

This cross-sectional study recruited a sample of 1131 parents of schoolchildren 8-18 years old and requested them to complete internationally accepted questionnaires for their children. Chi-squared test and logistic regression analysis were used to analyze the data (*p* < 0.05).

**Results:**

A majority (89.2%) of schoolchildren were bully victims. Physical bullying (48.9%) was the most common form of bullying. The youngest schoolchildren (8-11 years) and those who disliked school classes or neither liked nor hated them, as well as those who were truant from school, were more likely to be victims. In addition, those who had worse grades because of bullying and those who were very often bullied because of good grades or because they showed an interest in school were more likely to be victims. With regard to targeted physical features, teeth were the number one target, followed by the shape of the lips and strength, while tooth shape and color were the most common dentofacial targets, followed by anterior open bite and protruded anterior teeth. Boys and the youngest schoolchildren were more often subjected to bullying because of these features (*p* < 0.05).

**Conclusions:**

The prevalence of bullying, mainly in a physical form, was high among Saudi schoolchildren, with a negative influence on students' academic abilities. Problems related to teeth, in particular, which can be treated, were targets, mainly for boys and the youngest schoolchildren. More studies are required in Saudi Arabia to explore the issue further among schoolchildren themselves.

## 1. Introduction

Bullying victimization among schoolchildren is endemic [1]. A student is considered to be bullied or victimized when he or she is exposed, repeatedly and over time, to negative actions on the part of one or more other students [2]. Negative actions can be classified as direct (hitting, kicking, insults, and threats) or indirect (gossip, spreading of rumors, and social exclusion) forms of aggression that cause harm to the victim [3]. Countries across the globe have identified bullying as a leading adolescent health concern [4] which can occur in any setting but typically occurs at school or on the journey to and from school [5].

The prevalence of bullying victimization among schoolchildren varies greatly in literature, and several factors have been suggested as contributors including age, gender, and cultural factors [6, 7]. Several studies have reported that boys are more often subjected to bullying than girls, and those schoolchildren who are younger than 12 years of age are also more often subjected to bullying as compared to older schoolchildren [3, 6, 8, 9].

Craig et al. [6] compared the prevalence of bullying among 11- to 15-year-old schoolchildren across 40 countries and reported estimates ranging from 8.6% to 45.2% among boys and from 4.8% to 35.8% among girls. In the Middle East, the issue of bullying was addressed among schoolchildren 12 to 18 years of age in Jordan [10], Lebanon [11], Oman [12], and UAE [13]. Prevalence estimates in these countries ranged from 20.9% to 44.2%. In Saudi Arabia, AlBuhairan et al. [14] explored the topic in the capital city (Riyadh) and reported that all forms of bullying (verbal, physical, sexual, psychological/social, and cyberbullying) were present in the Saudi community. Cyberbullying is a new form of bullying where the aggressive or intentional act is carried out repeatedly and over time by a group or individuals, using electronic forms of contact, mainly through phones and the Internet, against a victim who cannot easily defend against it [15]. The absence of geographical and temporal limits worsens the condition further due to the difficulty in accessing the victims and the higher likelihood of victims to face constant harassment. A prevalence estimate of 26% was reported for bullying victimization among schoolchildren 12 to 18 years of age in Saudi Arabia [16].

Physical features such as weight have been linked with bullying; overweight schoolchildren were found to be more often subjected to bullying regardless of their gender [17]. In addition, dental features incited the creation of nicknames, harassment, and teasing among schoolchildren; comments about teeth were considered more hurtful than comments about height or weight [18–20].

The role of parents in identifying and addressing the issue of bullying in their children is important. Few studies pointed out that parents may be unaware that their children are being bullied [21]. However, this may not be the case with all populations. It has been observed that bullying often changes a child's behavior and is therefore very often detected by parents. In addition, parents of victims are commonly interested in reporting what has happened to their children [3, 22].

The issue of bullying victimization was never addressed from a broad perspective in the Kingdom of Saudi Arabia, including prevalence, forms, and influence of the condition on students' academic abilities and school attendance, as well as related sociodemographic, physical, and dentofacial features across a wide age range of schoolchildren. Therefore, the aim of the current study was threefold: first, to determine the prevalence of bullying, as well as its forms and associated sociodemographic factors; second, to determine the effect of bullying victimization on academic abilities and school attendance; and third, to determine associated physical and dentofacial features with the condition among Saudi schoolchildren aged 8 to 18 years old.

## 2. Materials and Methods

### 2.1. Study Population and Ethical Approval

This was a cross-sectional study conducted on a convenience sample of 1131 Saudi parents who were contacted through social networking sites (Facebook and Twitter). Parents were approached through social networking sites as these are very popular in Saudi Arabia. Chan et al. [23] also reported that the public was more comfortable sharing personal bullying experiences on publicly accessible social media forums such as Twitter.

All participating parents in the current study provided their written consent. Ethical approval was also obtained prior to the start of the study (reference number: EA/6041/2019). The inclusion criteria for the study were parents who were residing in any of the five geographical regions of Saudi Arabia and who spoke and understood the Arabic language; parents who had a healthy (with no chronic illness or congenital anomaly) schoolchild in the age range of 8-18 years who did not have an orthodontic appliance in situ; parents who provided their written consent to participate in the study; and parents who had an account on either Facebook or Twitter. In the current study, parents were the caregivers of the schoolchildren. They were their legal guardians.

### 2.2. Study Measures

Parents were sent a structured electronic questionnaire in the Arabic language during the period from December 2019 through March 2020.

The questionnaire was written initially in the English language; most of the questions used in the questionnaire were adopted from questionnaires used by Shaw et al. [24] and Al-Bitar et al. [10]. The questionnaire which was adopted by Al-Bitar et al. [10] and which most of the questionnaire questions were adopted from was in the Arabic language in a similar Arabic population (Jordan). Few questions were added by the authors in the current study to meet the study objectives. The questionnaire was translated into the Arabic language and then back into the English language to ensure accuracy. The English and Arabic versions were validated by two independent investigators to ensure the similarity of the content of both versions. The questionnaire consisted of 42 items, in the form of multiple-choice questions, was distributed in three parts, and was aimed at assessing (1) the sociodemographic profile of participants and their experience of bullying; (2) parental opinion about the effect of bullying on their child's feelings toward school and on school attendance, as well as bullying's perceived effect on academic performance; and (3) general physical characteristics and dentofacial features targeted in the victims of bullying as reported by parents. In this study, for those parents who reported having a bully victim, a question was added to part one of the questionnaire to identify the form of bullying that the child experienced. In addition, because parents answered the questionnaire for their children, a third answer option (do not know) was added to the questions that were in yes/no format. The exception was the question about whether the child was subjected to bullying in the past month, which was kept in yes/no format; parents who could not answer that question were asked to leave it blank. At the beginning of the questionnaire, a brief definition of bullying—the same definition adopted by Olweus [2]—was given, along with the objectives of the study and the consent statement. To avoid confusion and the difficulty in recalling information about several children, in the case of parents having more than one schoolchild who matched the inclusion criteria, parents were requested to select one of their children and answer the questionnaire according to that child's age and gender. To ensure content and context validity, the questionnaire was piloted among a sample of 31 parents to ensure that all questions were clear. Neither the questions nor the answers were modified following the pilot study, and the pilot sample was excluded from the study's main sample.

### 2.3. Data Analysis

Statistical analysis of the data was conducted using the SPSS software (version 22.0; SPSS, Chicago, Ill). Descriptive statistics were obtained, and a chi-squared test (univariate approach) was applied to detect any significant differences between responses according to bullying status in parts one and two of the questionnaire. Descriptive statistics and a chi-squared test were also used to compare responses according to the age group and gender of the schoolchildren in part three of the questionnaire. Binary logistic regression (multivariate approach) was further used to ascertain the sociodemographic variables and the variables related to students' academic abilities and school attendance, which were associated with being a bully victim. Probability values of *p* < 0.05 were considered statistically significant throughout.

## 3. Results

A total of 1131 questionnaires were shared with parents through Facebook and Twitter. Of these, 500 questionnaires were shared through Facebook and 631 questionnaires were shared through Twitter. A total of 103 questionnaires were not answered completely and were consequently excluded. The overall response rate was 90.9%. The final sample contained 1028 parents (450 fathers and 578 mothers) who answered questionnaires for 1028 schoolchildren. These represented 1.2% of schoolchildren of that age range in Saudi Arabia as estimated in 2020 [25].

Table 1 provides the frequencies, percentages, and differences (by bullying status) for the study population according to sociodemographic background and bullying experience as perceived by parents. Of 1028 schoolchildren, 917 (89.2%) were bully victims, while only 14.1% were bullies. The majority (87.3%) of bully victims were bullied by a group of 2-5 students. Physical bullying, in the form of fights with other students, was the most common form (48.9%), followed by a combination of more than one bullying form (21.3%), verbal bullying (21%), and cyberbullying (5.1%). The majority (73.7%) of bully victims were within the age group of 8-11 years, with around two-thirds of them being boys (61.7%). In addition, the majorities of bully victims were from public schools (77.7%) and had nicknames (82.1%) that were used primarily by relatives (49.3%) and peers (23.8%). These nicknames were disliked by around half of the bully victims (54.2%). A chi-squared test revealed a significant association between bullying victimization and educational level and occupation of the parent, family monthly income, residence region in Saudi Arabia, age group and gender of the schoolchild, having a nickname, feeling about the nickname, and acting as a bully (*p* < 0.05).

A description of feelings toward school and the relationship to academic performance is shown in Table 2. The great majority (83%) of bully victims hated their school classes and were truant from school because of bullying (82.8%), as reported by parents. Almost all parents (96.8%) believed that bullying negatively affected their children's school grades and that having good grades or showing interest in school were reasons for bullying victimization (92.1%). There were statistically significant differences between bullying victimization and feelings about school classes, truancy from school, effect on school grades, and extent of bullying victimization because of good grades or showing an interest in school (*p* < 0.001).

The results of the first two parts of the questionnaire were further adjusted using binary logistic regression analysis to ascertain the factors associated with being a bully victim. The factors that were entered into the regression model included those factors that dictated the children's sociodemographic profile and the effect of bullying victimization on their academic abilities and school attendance. These were education level and occupation of the parents, family monthly income, residence region in the kingdom, age group and gender of the schoolchild, feelings inside school classes, being truant from school, effect on school grades, and extent of bullying victimization because of good grades or showing an interest in school. The factors associated with bullying victimization according to the multivariate analysis are shown in Table 3. They included age group of the schoolchildren, feelings inside class, being truant from school, effect on school grades, and extent of bullying victimization because of good grades or showing an interest in school (*p* < 0.05). The rest of the factors were not associated with bullying victimization (*p* > 0.05).

Compared to schoolchildren who were 8-11 years old, those who were 16-18 years old had 5.52 times (95% confidence interval (CI): 0.072-0.456) lower odds of being bully victims. In addition, those who hated their school classes, or who neither liked nor hated them, had 8.97 and 2.04 times (CI: 2.984-26.958 and 1.067-3.892, respectively) higher odds of being bully victims than those who liked their school classes. Compared to schoolchildren who were truant from school, those who were not or were unsure about the issue of truancy from school had 20 and 7.7 times (CI: 0.018-0.137 and 0.038-0.447, respectively) lower odds of being bully victims. Moreover, schoolchildren whose school grades were affected by bullying either a lot or a little had 3.74 and 2.86 times (CI: 1.852-7.550 and 1.246-6.559, respectively) higher odds of being bully victims than those who experienced no effect on their school grades. On the other hand, compared to schoolchildren who reported to be subjected a lot to bullying because of good grades or showing an interest in school, those who reported not being subjected to bullying at all had 3.94 times (CI: 0.076-0.851) lower odds of being bully victims.

**Figure 1:**
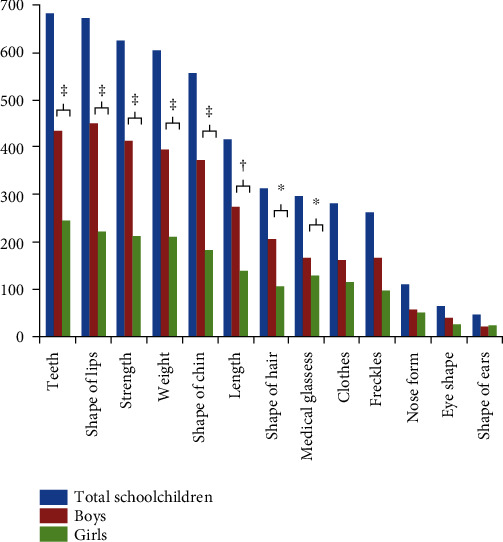
Frequency distribution of physical features reported as targets for victimization according to gender. ^∗^*p* < 0.05, ^†^*p* < 0.01, and ^‡^*p* < 0.001.

**Figure 2:**
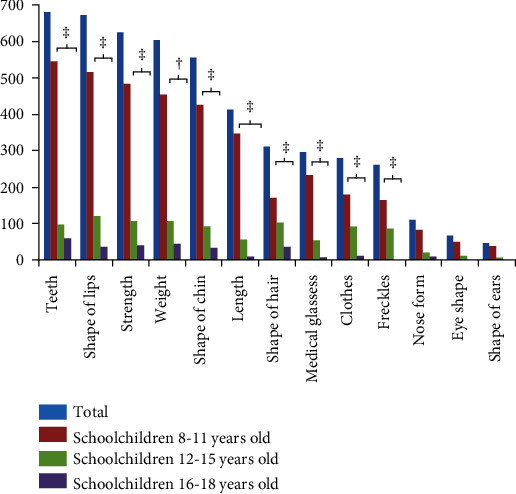
Frequency distribution of physical features reported as targets for victimization according to age group. ^∗^*p* < 0.05, ^†^*p* < 0.01, and ^‡^*p* < 0.001.

General physical characteristics were targeted in the victims of bullying. Targeted physical features according to gender and age group are shown in Figures 1 and 2. The top five physical features reported as being targets of bullies were, in descending order, as follows: teeth > shape of lips > strength > weight > shape of chin. Significantly more boys were bully victims because of targets including teeth, shape of lips, strength, weight, shape of chin, length, shape of hair, and medical glasses as compared to girls (*p* < 0.05), while significantly more schoolchildren from the youngest age group (8-11 years) were bully victims because of the aforementioned targets, as well as because of clothes and freckles, as compared to older age groups (*p* < 0.05).

**Figure 3:**
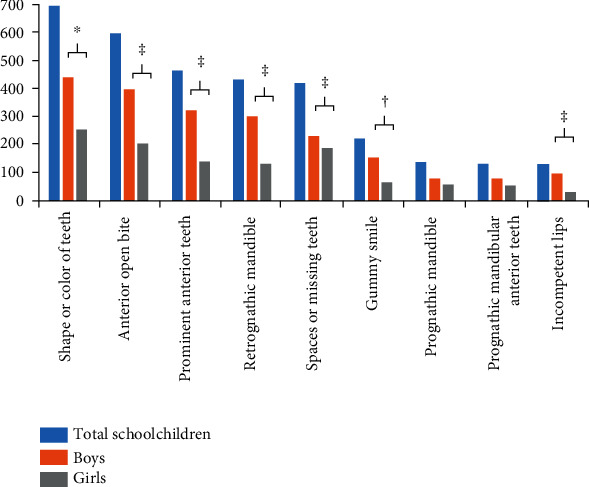
Frequency distribution of dentofacial features reported as targets for victimization according to gender. a^∗^*p* < 0.05, ^†^*p* < 0.01, and ^‡^*p* < 0.001.

**Figure 4:**
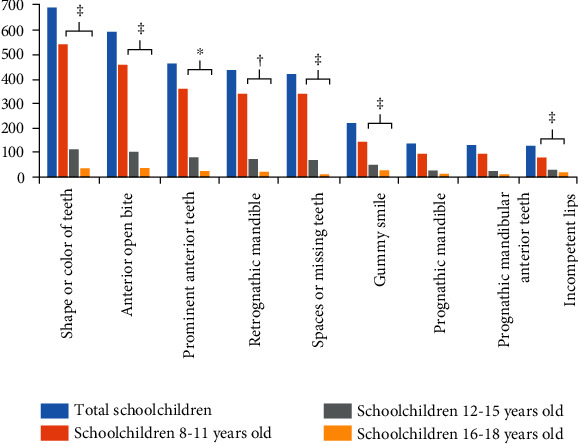
Frequency distribution of dentofacial features reported as targets for victimization according to age group. ^∗^*p* < 0.05, ^†^*p* < 0.01, and ^‡^*p* < 0.001.

Dentofacial features were targeted in the victims of bullying. The dentofacial features that were identified as targets by bullies according to gender and age group are shown in Figures 3 and 4. The top six features in descending order were shape or color of teeth > anterior open bite > prominent anterior teeth > retrognathic mandible > spacing between teeth/missing teeth > and gummy smile. Significantly more boys were bully victims because of these dentofacial targets as compared to girls. In addition, significantly more boys were bully victims because of incompetent lips. Significantly more children from the youngest age group (8-11 years) were bully victims because of these seven dentofacial features as compared to older age groups (*p* < 0.05).

## 4. Discussion

The current study is the first to investigate the issue of bullying victimization among a wide age range of Saudi schoolchildren; its forms, as well as effect on academic abilities; and associated factors, particularly physical, and dentofacial features. It was decided to let parents answer for their children, despite that the best approach is to let schoolchildren, particularly adolescents, answer for themselves as they can understand the questions and answer on their own, to standardize the source of information across all age groups and enable comparisons with the youngest schoolchildren (8-11 years), who are less likely to have developed sufficient cognitive skills which can enable them to understand the questions fully on their own, and give reliable answers as compared to adolescents [26]. Consequently, it would be inappropriate to let the parents answer on behalf of the youngest schoolchildren and let adolescents answer for themselves. The same approach was followed in the Nordic countries [3, 22].

In the current study, a questionnaire was preferred over interviews as an assessment tool for purposes of enabling comparisons with different countries that used questionnaires because of the sensitivity of the topic [8, 10, 27]. It was previously reported that denial of the bullying condition, which should be embarrassing when confronted about, would be less in questionnaires as compared to direct interviews [10]. The questionnaire began with a clear definition of bullying, and the forms of bullying included examples to minimize the subjectivity and bias that may result from self-perceptions of bullying victimization [28]. Particular attention was paid to the inclusion of sociodemographic information other than the child's age group to maximize our ability to make inferences about specific subsets of the population.

The current study has shown that bullying victimization is highly prevalent in Saudi schools. The estimated prevalence (89.2%) could be the highest among studies carried out in the Middle East and across the globe [6, 8, 10–12, 27]. However, when the prevalence of the condition is considered separately for each age group, the prevalence estimate for children younger than 12 years of age in the current study (73.7%) was just slightly lower than that reported in Jordan (80%) [8]. In addition, bullying victimization prevalence among those 12-18 years of age in the current study was 26.5%, which is similar to that reported previously in Saudi Arabia (26%) [16]. Among the whole sample, 50 parents were not able to classify the bullying status of their children; consequently, their questionnaires were excluded. This indicates that almost all parents correctly classified the bullying status of their children, and reflects good communication between Saudi parents and their schoolchildren.

The physical form of bullying, mostly in the form of fights with other students, and a combination of more than one form of bullying, such as verbal insults and social rejection altogether or verbal insults and cyberbullying, were the most common forms in the current study. These findings are in contrast to previous reports in other countries, where verbal bullying and spreading rumors were the most common forms [7, 10, 11]. Physical violence was addressed as a major and prevalent issue among Saudi schoolchildren 12-18 years old [16]. In addition to physical violence, bullying that can be in multiple forms seems to be an emerging issue that has never been addressed and that warrants attention.

Among the sociodemographic factors assessed in the current study, only age group was associated with bullying victimization. The youngest age group of schoolchildren (8-11 years) had a significantly higher likelihood of being bully victims as compared to the older age groups. This finding is important because it indicates that most children who were bullied were younger than 12 years old. This finding is consistent with the findings from previous studies [3, 8, 9].

In this study, bullying victimization negatively affected academic performance and school attendance; the factors associated with a greater likelihood of bullying victimization included hating or neither liking nor disking school classes, being truant from school, and having good grades or showing an interest in school. In addition, physical and dentofacial features were reported as targets for victimization. The most common physical features targeted were teeth, shape of lips, strength, weight, and shape of the chin. These physical features were also reported to be targets by bullies in other studies [10, 17, 27]. On the other hand, shape or color of teeth, anterior open bite, prominent anterior teeth, retrognathic mandible, and spacing or missing teeth were the most targeted dentofacial features among bully victims. Similar findings were reported in previous studies [10, 27]. In addition, boys and the youngest schoolchildren (8-11 years old) suffered bullying because of physical and dentofacial features the most. This indicates that malocclusal traits can be a source of trouble for boys just as they can be for girls, if not even more so, which consequently highlights the importance of having a qualified health professional at Saudi schools to perform screening for the physical and dentofacial features which were reported to be targets for bullying in the current study, as most of these features, particularly those related to teeth, can be treated when identified [27]. Schoolchildren identified as having the physical or dentofacial features reported as being targets of bullies can be referred to relevant specialists for further assessment of their problem and arrangement for treatment if that is deemed necessary and possible. The majority of involved schoolchildren in the current study are from high-income families and their parents are well-educated (university degree holders). Better economic and social conditions should improve access to the treatment of oral problems in general [29].

Several limitations of the current study should be noted; the first limitation is that the current study was cross-sectional in design and based on parental reported questionnaires. This can be insufficient to ascertain a causal relationship between victimization and health problems. In addition, a risk of biased reporting still exists, particularly for parents who answered for older age groups. It is also likely that parents who had bullied schoolchildren were keen to participate and answer the questionnaires more than those parents who did not have such experience. It is important to mention, however, that out of all distributed questionnaires (1131) in the present study, only 103 questionnaires were excluded; consequently, even if those parents whose questionnaires were excluded reported not to have a bullied child, the conclusion that a high prevalence of the issue is present will not probably change. The second limitation is that use of a convenience sample may not represent the entire population, despite that different social, economic, and educational diversities were included in the studied population, as around two-thirds of the parents who returned the questionnaires completed were well-educated and had high monthly income and they answered the questionnaire for their sons; consequently, this may limit the generalizability of the results, particularly concerning the association of the sociodemographic factors with bullying victimization. However, the results of the present study can still provide baseline data that need to be further explored in future studies conducted on representative samples of, preferably, Saudi schoolchildren themselves. A third limitation would be that we were unable to identify those who were acting as bullies, as just less than half of the parents (45%) did not know whether or not their children acted as bullies. It is important to identify the bullies and the victims and to determine whether the bullies are in higher classes than the bully victims or in the same class. Gangs of bully students should be identified. Parents, schoolchildren, and all school staff should be involved in fighting against bullying. As parents were aware that the issue exists, it is important to encourage parents who reported having a bullied child to report the issue to school officials to implement effective antibullying interventions. The role of the school counselor and teachers can be improved; for example, school officials should focus on having special classes with the schoolchildren, particularly the youngest schoolchildren (8-11 years), to educate them about the issue of bullying victimization and that human beings should be valued and respected regardless of how their physical features look like. The lessons and themes carried out in these classes can involve discussion, group work, short films about bullying, and role-play exercises of bullies and victims [30]. It is also better to have a health professional in each school, qualified to examine schoolchildren and identify physical and dentofacial targets for bullying, when deemed necessary. Identified bully victims, who after the screening, prove to have any of the physical or dentofacial features known to be targets for bullying can be referred for concerned specialists. Until further studies on the topic are conducted in Saudi Arabia, it is better to ensure the presence of teachers during school break times to discourage students from misbehaving.

## 5. Conclusion

Bullying victimization, mainly in a physical form, seems to be a serious phenomenon in Saudi Arabia, with a negative influence on students' academic abilities and school attendance. A prevalence estimate of as high as 89.2% was found, particularly among young schoolchildren 8-11 years of age (73.2%). Physical and dentofacial features were found to be associated factors, particularly among boys and young schoolchildren. Problems related to teeth, in particular, which very often can be treated, were targets for bullying. More studies are needed in Saudi Arabia to explore the issue further among schoolchildren themselves.

## Figures and Tables

**Table 1:** Sociodemograhic characteristics of study participants and association with bullying (*n* = 1028).

Variables	Category	*N*	%	Bully victims			*p* *value*°
				No (*n* = 111)	Yes (*n* = 917)		
				*n*	*n*	%	
Educational level of parent	Did not go to school	6	0.6	0	6	0.7	0.005^∗^
	Primary	8	0.8	2	6	0.7	
	Intermediate	27	2.6	1	26	2.8	
	Secondary	247	24.0	13	234	25.5	
	University	740	72.0	95	645	70.3	
Occupation of parent	Health sector	197	19.2	13	184	20.1	0.034^∗^
	Education sector	634	61.7	70	564	61.5	
	Other	15	1.5	4	11	1.2	
	Unemployed	182	17.7	24	158	17.2	
Family monthly income	<1680 USD	59	5.7	15	44	4.8	<0.001^∗^
	1681-3200 USD	188	18.3	31	158	17.2	
	>3200 USD	781	76.0	66	715	78.0	
Residence region in the kingdom	Central	408	39.7	73	335	39.7	<0.001^∗^
	Eastern	167	16.2	11	156	16.2	
	Western	155	15.1	12	143	15.1	
	Northern	153	14.9	5	148	14.9	
	Southern	145	14.1	10	135	14.1	
Age group of child	8-11	734	71.5	59	675	73.7	<0.001^∗^
	12-15	220	21.4	28	192	21.0	
	16-18	73	7.1	24	49	5.3	
Gender of child	Boy	614	60.0	50	564	61.7	0.001^∗^
	Girl	410	40.0	60	350	38.3	
School sector	Public	800	78.0	90	710	77.7	0.246
	Private	225	22.0	21	204	22.3	
Child has nickname	Yes	778	75.7	25	753	82.1	<0.001^∗^
	No	177	17.2	81	96	10.5	
	Do not know	73	7.1	5	68	7.4	
Feeling about nickname	Like	145	17.3	13	132	16.5	<0.001^∗^
	Neutral	248	30.6	13	235	30.3	
	Dislike	445	53.1	10	435	54.2	
Child acted as a bully	Yes	146	13.6	7	129	14.1	<0.001^∗^
	No	476	44.2	88	373	40.7	
	Do not know	454	42.2	16	415	45.3	

°According to chi-squared and Fisher's exact tests, ∗ indicates statistically significant difference (*p* < 0.05).

**Table 2:** Feelings toward school and relationship of academic performance and bullying.

Variables	Category	*n*	%	Bully victim			*p* *value*°
				No (*n* = 111)	Yes (*n* = 917)		
				*n*	*n*	%	
Inside class	Like	115	11.2	59	56	6.1	<0.001^∗^
	Ambivalent	144	14.0	44	100	10.9	
	Hate	769	74.8	8	761	83.0	
Outside class	Like	695	67.6	80	615	67.1	0.559
	Ambivalent	314	31.5	30	285	31.1	
	Hate	19	1.8	2	17	1.9	
Absence from school	Yes	767	74.6	8	759	82.8	<0.001^∗^
	No	184	17.9	94	90	9.8	
	Do not know	77	7.5	9	68	7.4	
Effect on school grades	Significant effect	531	51.7	57	474	51.7	<0.001^∗^
	Little effect	444	43.2	31	414	45.1	
	No effect	53	5.2	24	30	3.2	
Times bullied because of interest in school or good grades	A lot	376	36.6	6	370	40.3	<0.001^∗^
	A little	509	49.5	34	475	51.8	
	Not at all	143	13.9	71	72	7.9	

°According to chi-squared and Fisher's exact tests, ∗ indicates statistically significant difference (*p* < 0.05).

**Table 3:** Logistic regression analysis of factors associated with bulling victimization.

Independent variables	Group	*B*	S.E.	Odds ratio	95% confidence interval		Sig.
					Lower	Upper	
Age group of child	*8-11^reference^*						
	12-15	-0.525	0.361	0.592	0.292	1.201	0.146
	16-18	-1.709	0.471	0.181	0.072	0.456	<0.001^∗^
Feelings inside class	*Like^reference^*						
	Ambivalent	0.712	0.330	2.038	1.067	3.892	0.031^∗^
	Hate	2.194	0.562	8.969	2.984	26.958	<0.001^∗^
Absence from school	*Yes^reference^*						
	No	-2.993	0.513	0.050	0.018	0.137	<0.001^∗^
	Do not know	-2.043	0.631	0.130	0.038	0.447	0.001^∗^
Effect on school grades	Significant effect	1.319	0.359	3.739	1.852	7.550	<0.001^∗^
	Little effect	1.050	0.424	2.859	1.246	6.559	0.013^∗^
	*No effect^reference^*						
Extent of bullying because of interest in school or good grades	*A lot^reference^*						
	A little	-0.380	0.588	0.684	0.216	2.164	0.518
	Not at all	-1.369	0.616	0.254	0.076	0.851	0.026^∗^

Hosmer and Lemeshow goodness-of-fit test (*p* *value* = 0.653), ∗ indicates statistically significant difference.

## Data Availability

The data used to support the findings of this study are available from the corresponding author upon request.
